# Executive Function Is Selectively Impaired in Old Age Bipolar Depression

**DOI:** 10.3389/fpsyg.2017.00194

**Published:** 2017-02-13

**Authors:** Leonardo Caixeta, Vânia L. D. Soares, Renata T. Vieira, Cândida D. Soares, Victor Caixeta, Sandra B. Ferreira, Tales A. Aversi-Ferreira

**Affiliations:** ^1^Bipolar Disorder Unit, Hospital das Clínicas, School of Medicine, Federal University of GoiásGoiania, Brazil; ^2^Unit of Neuropsychiatry, Neuropsychology and Behavior Neurology (UNCO), Federal University of GoiásGoiania, Brazil; ^3^Federal University of AlfenasAlfenas, Brazil; ^4^System Emotional Science, School of Medicine and Pharmaceutical Sciences, University of ToyamaToyama, Japan

**Keywords:** aging, bipolar disorder, elderly patients, executive dysfunctions, neuropsychology

## Abstract

**Background:** Little is known about the cognitive signature of bipolar disorder (BD) in elderly brains. The neuropsychological features of depressive elderly with early-onset BD are largely unknown. This issue is relevant because cognitive impairment can produce an additional impact on the already compromised functionality of elderly with BD. The aim of this study is to assess executive functions (EFs) in the depressive phase of elderly outpatients with early-onset BD.

**Methods:** Forty-nine elderly outpatients with early-onset BD were assessed with several neuropsychological tests for EF in the depressive phase of the disorder.

**Results:** Executive dysfunction is very common in old age bipolar depression. Thirteen patients (26.5%) had a pseudodementia presentation. The worst performances were observed in the following tests: Trail Making B, Stroop Test 3, Backward Digit Span and Wisconsin Card Sorting Test.

**Conclusion:** Executive dysfunction profile in elderly BD is complex and heterogeneous, but most cases display difficulties in working memory, inhibitory control, mental flexibility, and information processing speed. The performance of elderly with bipolar depression in executive assessment can be divided into two main categories: (1) Single EF domain impairment; and (2) Multiple EF domain impairment with or without a pseudodementia syndrome. Executive dysfunction in old age bipolar depression may be explained by lack of sufficient mental energy to run those cognitive processes that require larger amounts of effort to be performed.

## Introduction

Bipolar disorder (BD) in the elderly is a growing public health concern and a major cause of disability (Sajatovic et al., 2015). Despite considerable knowledge accumulated in recent decades points to the presence of cognitive impairment in BD (Tsitsipa and Fountoulakis, 2015), few consistent data exist on the neuropsychology of old age patients with BD. Studies with very selective samples and multiple age groups suggest that higher age in BD is associated with greater neurocognitive deficits (Kessler et al., 2013). Some authors state that elderly patients with BD have significant cognitive disabilities and that BD with late onset is associated with more severe cognitive impairment than early-onset BD (Schouws et al., 2007, 2009), but all these studies were done in euthymic BD. There is a lack of knowledge especially in relation to the neuropsychology of bipolar depression in late life (Rise et al., 2016).

Little is known about the cognitive signature of BD in elderly brains. This issue is relevant because cognitive impairment can produce an additional impact on the already compromised functionality of elderly with BD (Rise et al., 2016). Besides that, the identification of specific cognitive profiles in BD can perhaps help differentiate subgroups and pave the way to the identification of possible endophenotypes (Volkert et al., 2016). Finally, a comprehensive mapping of cognitive domains impaired in BD can add some insights into the physiopathology of the disorder and the many mechanisms by which cognitive symptoms may relate to behavior alterations. For instance, in the young adult it is known that cognitive deficits in BD are directly related to the daily losses presented by these patients in social adaptation or even in suicide behavior (Malloy-Diniz et al., 2009).

Among the most disputed issues in contemporary cognitive neuropsychiatry is the deterioration of executive functions (EF) and the neurocircuitry disruption that support these disabilities. EFs impact affective-emotional, motivational, and social skills. Executive dysfunction may represent an important contributor to the cognitive, functional and social impairments usually observed in adults (Tsitsipa and Fountoulakis, 2015) and elderly (Schouws et al., 2009) with BD.

The aim of this study is to assess EFs in the depressive phase of elderly outpatients with early-onset BD.

## Materials and Methods

### Participants

We have selected 49 consecutive elderly outpatients diagnosed by the same senior psychiatrist (LC) as a BD according to DSM-5 criteria, from the BD program in a university hospital (Hospital das Clínicas at the Federal University of Goiás) in Central Brazil, from August 2013 to 2015. All subjects provided written informed consent, as required by the ethical committee of Federal University of Goiás. The hospital’s catchment area involves a population of approximately 1500 elderly. We calculated a sample size of 50.33 patients with a sampling error of approximately 2.13% at a 95% confidence level. Originally, 157 elderly bipolar patients were enrolled in the service. Of these, 91 were eliminated by the exclusion criteria (44 patients with late-onset BD – type VI; 42 with dementia; 3 alcoholics; and 2 with psychostimulant use). Thus, 66 patients were eligible for the study, but 17 refused or could not undergo neuropsychological evaluation, leaving, therefore, 49 patients to compose the definitive sample.

Inclusion criteria were: age 60–90 years old; the onset of BD prior to the age of 40 years (definition of ‘early-onset’ BD [Schouws et al., 2009]); any ethnicity; portuguese speaking Brazilian outpatients; followed at least during 1 year in our service with serial (monthly) psychiatric assessment in order to rule out primary dementia cases. We have included any degree of cognitive and functional impairment. We have excluded from our search elderly bipolar patients who began their disease late in life (BD type VI), since this subtype has a strong association with neurological disease and structural damage to the brain (Ng et al., 2008), what could interfere in the neuropsychological performance. The same senior psychiatrist (LC) made the diagnosis of BD and assessed the diagnosis for the depressive phase of BD.

According to international recommendations (Burdick et al., 2015), it was excluded subjects taking high-dose anticholinergics, topiramate, clozapine, tricyclics, benzodiazepines, psycho-stimulants, and those who had electroconvulsivetherapy within past 6 months. Medication titration could not be done in the week of neuropsychological assessment. Patients with general clinical conditions which could impact cognitive performance were also not included in our sample: delirium, untreated hypothyroidism, severe anemia, AIDS, any kind of encephalopathy.

### Laboratory Testing and Neuroimaging

All patients were submitted to laboratory screening and neuroimaging exams according international recommendations (Knopman et al., 2001) in order to rule out systemic or neurological causes of cognitive impairment and primary dementias with structural brain damage.

### Neuropsychological Assessment

Neuropsychological assessment of the patients was carried out by three senior clinical neuropsychologists (VLDS, CDS, SBF).

Patients were evaluated during the depressive phase. Total assessment was divided into three sessions of 30 min each within a period of 3 days in order to prevent mental fatigue. All patients were submitted to the most frequently used tests for assessing EFs in aging (Faria et al., 2015), consisting of subtests from standard test batteries validated for Portuguese use in Brazilian patients: (1) Trail Making Test Form A (TMT-A): attention, information processing speed; (2) Trail Making Test Form B (TMT-B): mental flexibility, information processing speed; (3) Stroop Test: inhibitory control; (4) Digits Forward and Backward subtests (WAIS-R): working memory; (5) Wisconsin Card Sorting Test (WCST): mental flexibility; (6) Verbal Fluency Test (Animals category): verbal fluency and information processing speed; (7) Verbal Fluency or Letter Fluency Test – FAS: verbal fluency, information processing speed and active search for specific information in memory.

### Statistical Analysis

Clinical data were statistically analyzed using conventional descriptive methods. Quantitative variables were described as means and standard deviations, while categorical variables were described using frequency and percentages.

When describing neuropsychological performance in depressive bipolar elderly, we transformed raw scores for all individual neuropsychological tests (except WCST) into *Z*-scores using the distribution of the older adult controls’ database (i.e., controls’ performance on any test has mean of 0 and SD of 1). For WCST was used *T*-score; scores were considered abnormal based on Brazilian norms and validation of these instruments in Brazilian population (Zimmermann et al., 2015).

## Results

The mean age of BD patients was 69.1 years (60–88, **±**7.0). Most patients were female (*n* = 31; 62.2%). The mean schooling was 10.8 years (**±**4.2).

**Table 1** shows the descriptive analysis (mean, standard deviation) of the neuropsychological assessment.

**Table 1:** Statistical description of executive function tests in elderly with bipolar depression (*n* = 49).

Tests	Minimum	Maximum	Mean	Standard deviation
Stroop Test 1	-6.05	6.24	1.17	2.30
Stroop Test 2	-0.02	7.52	2.02	2.01
Stroop Test 3	-5.19	1.55	-1.05	1.42
Errors Stroop 1	0	1.00	0.04	0.20
Errors Stroop 2	0	6.00	0.23	0.98
Errors Stroop 3	0	15.00	1.79	3.17
TMT-A	-8.08	8.05	1.30	3.38
TMT-A errors	0	4.00	0.19	0.77
TMT-B	-10.44	1.06	-2.02	2.40
TMT-B errors	0	26.00	8.84	8.44
VF Digit Span	-1.85	1.34	-0.55	0.48
VB Digit Span	-2.54	0.10	-1.17	0.55
VFT – Animals category	-2.39	1.66	-0.86	0.81
VFT – FAS	-2.31	1.89	-0.69	1.00
WCST	15	49.00	24.42	9.27

*TMT, Trail Making Test; VF Digit Span, Visual Forward Digit Span; VB Digit Span, Visual Backward Digit Span; VFT, Verbal Fluency Test; WCST, Wisconsin Card Sorting Test. For all tests (except WCST) was used *Z*-score. For WCST was used *T*-score*.

Of patients, 28 (57.1%) scored -2SDs (moderate impairment) on at least one EF test measure, and 49 (100%) scored lowest than -1SD on at least one EF test measure or, in other words, none of the patients had a normal performance in all EF tests.

Forty patients (81.6% of total sample) presented a more extensive and severe dysexecutive profile, involving many EFs. From these, thirteen patients (26.5%) had a pseudodementia presentation.

Nine patients (18.3%) had a mild executive dysfunction (affecting, for example, only one executive domain, such as working memory or mental flexibility).

The mean of WCST (24.4) according *T*-score is in the diagnostic category of “moderately-to-severely impaired range” (Heaton et al., 1993). No patient was able to complete all categories of WCST perfectly.

Twenty-three patients (46.9%) had scores lowest than -1SD on VFT-animals and 22 (44.8%) on FAS. On average, patients had difficulty in verbal fluency tasks as indicated by negative *Z*-scores, but the mean does not exceed the adopted *Z*-score cutoff. On average, performance on VFT (Animals category) was worse than FAS (-0.86 versus -0.69, respectively).

## Discussion

Our data support the notion that executive dysfunction is very common in elderly with BD, since all the sample had performance below average in at least one EF test. Moreover, we report new data concerning executive dysfunction in BD elderly outpatients in the depressive phase, since studies are focused on euthymic or manic elderly (Young et al., 2006; Schouws et al., 2007, 2009; Samamé et al., 2013). It is not clear whether bipolar and unipolar old age depression have similar or different cognitive profiles, but our data, when compared to the cognitive profile described in the literature for unipolar depression in the elderly (Dybedal et al., 2013; Pantzar et al., 2014), show similarity in executive dysfunction between both forms of depression. Xu et al. (2012) studying adults state that bipolar and unipolar patients have a similar pattern of cognitive impairment during the state of acute depressive episode.

It is remarkably difficult to make definite statements about the neuropsychology of BD, since it depends on the BD type, age considered, medications in use, and mood state at the time of neuropsychological assessment (Tsitsipa and Fountoulakis, 2015). Even in a relatively homogeneous sample as ours, in which we have specifically arranged elderly, BD patients, in the depressive phase, the cognitive profile is not uniform. Notwithstanding all the sample presented with some degree of executive dysfunction, it was found a large span of executive performance among subjects, as indicated by the high variability between minimum and maximum scores, and varying from mild executive dysfunction (affecting, for example, only one executive domain, such as selective attention or working memory), until a more extensive and severe dysexecutive profile (involving many EFs, such as mental flexibility, mental engagement, self-control, self-monitoring, many attention domains and working memory). Therefore, BD elderly patients can indeed reach a level of cognitive dysfunction in their depressive phase compatible with a dementia syndrome (depressive pseudodementia). Based on our data, we can divide patients’ performance in neuropsychological assessment into two main categories: (1) Single EF domain impairment; and (2) Multiple EF domain impairment. The second one can present with or without a pseudodementia syndrome.

One of the most impaired functions in the elderly with BD in our sample was mental flexibility, assessed by TMT-B and WCST. These two tests also recruit working memory, selective attention, mental engagement and are sensitive to impulsive behavior (Faria et al., 2015). As these functions are related to the dorsolateral prefrontal areas in the brain, we can assume that in depressive phase of BD elderly the neural circuits involving this topography are somewhat affected. In fact, some authors (Brooks et al., 2010) have found dorsolateral prefrontal hypometabolism using positron emission tomography associated with impaired performance on executive tasks among older adults with BD.

Inhibitory control is a core member of the EFs and generally refers to the capacity to actively inhibit or delay a dominant response to achieve a goal. Inhibitory control is impaired in BD elderly as showed by poor performance in Stroop Test 3 seen in our sample. The ability to inhibit an automatic behavior and perform a controlled behavior is associated to ventral-fronto-striatal circuitry (Durston et al., 2002), therefore we can suggest that this network is somewhat impaired in the depressive phase of BD. Penfold et al. (2015) also found inferior prefrontal hypoactivation (using MRI) in medication-free adults with bipolar depression during response inhibition test. The weakening of inhibitory control can contribute to some aspects of social and functional impairment seen in BD, and can explain in part how depressive BD meet the day-to-day demands of conflict, delay, and compliance challenges.

Information processing speed is impaired in our sample of old age bipolar depression as suggested by TMT-B poor performances. Processing speed was also one of the most impaired cognitive functions in many studies dealing with BD in adults (Xu et al., 2012; Kessler et al., 2013). In fact, some authors (Volkert et al., 2016) suggest that reduced psychomotor speed could serve as a potential endophenotype for BD.

Working memory was also importantly impaired in our sample, as demonstrated by Digits Backward subtest of WAIS-R. Information storage by verbal working memory (measured in Digits Forward) and verbal content processing in working memory (measured in Digits Backward) are EFs highly sensitive to depressive mood states.

Verbal fluency was not importantly impaired in most cases of our sample, suggesting that the neural circuits involved in this cognitive function generally are not impacted by depressive state in bipolar elderly. In adults this finding might be different, since some authors have found important deficits in verbal fluency among depressive bipolar patients (Xu et al., 2012).

Executive dysfunction in elderly in the depressive phase of BD may be associated with lack of sufficient mental energy to run those cognitive processes that require larger amounts of effort to be performed. For instance, patients in our sample had more difficult in TMT-B than TMT-A, and in Stroop Test 3 than the other phases, suggesting that, as cognitive demand increases, their cognitive reserve is overpowered and, as a consequence, they cannot keep up with the growing executive demands. According to this hypothesis, this cognitive impairment would be reversible, returning to normal cognitive functioning as soon as patients switch their depressive state into an euthymic (normal mood) phase. Follow-up studies are required to test if this executive dysfunction in BD elderly is really transitory and completely dependent upon the affective phase of BD. One study has addressed cognitive outcomes in longitudinal assessment of old age euthymic BD (type I and II) and concluded that they did not exhibit accelerated cognitive decline over 2 years (Gildengers et al., 2012). Another study performs a systematic review about the neurocognitive dysfunction in BD and concluded that cognitive dysfunction is an enduring component and represents a core primary characteristic of BD, rather than being secondary to the mood state or medication, but they add that this core deficit can be confounded (either increased or attenuated) by the disease phase, in other words, depression may produce a temporary increase in the total amount of cognitive deficits seen in BD, which is in accordance with our view (Tsitsipa and Fountoulakis, 2015).

As a methodological limitation of our study, despite having restricted our sample only to the depressive phase of BD, this mood state in itself is very heterogeneous, varying from mild to severe depression. Future studies must try to control this variable.

In summary, executive dysfunction was very common in BD elderly patients in our sample, notwithstanding it was not severe in most of the cases. BD seems to contribute with additional burden to the aging process in the brain. Trail Making B, Stroop Test 3, Backward Digit Span and WCST represent a useful selection of tests sensitive to the specific executive dysfunction presented in old age bipolar depression. These tests seem more suitable than global screening cognitive measures frequently used in samples of elderly patients with cognitive impairment (e.g., the Mini-Mental State Examination; MMSE), which are likely not applicable in BD context due to low sensitivity (Burdick et al., 2015). The general pattern of specific executive deficits presented by our sample may suggest anomalous prefrontal-subcortical activation in elderly with BD. These cognitive findings can help understand some social and functional impairments seen in this disorder and indicate therapeutic targets as well as insights into its neurophysiopathology.

## Author Contributions

LC conception or design of the work; data collection; data analysis and interpretation; drafting the article. VS data collection; data analysis and interpretation. CS data collection; data analysis and interpretation. RV data collection; data analysis and interpretation; drafting the article. SF data collection; data analysis and interpretation. VC data collection; data analysis and interpretation. TA-F data collection; data analysis and interpretation; drafting the article.

## Conflict of Interest Statement

The authors declare that the research was conducted in the absence of any commercial or financial relationships that could be construed as a potential conflict of interest. The reviewer MAB and handling Editor declared their shared affiliation, and the handling Editor states that the process nevertheless met the standards of a fair and objective review.
